# Fecal Excretion of *Mycobacterium leprae*, Burkina Faso

**DOI:** 10.3201/eid2709.211262

**Published:** 2021-09

**Authors:** Ajay Vir Singh, Rajbala Yadav, Harpreet Singh Pawar, Devendra Singh Chauhan

**Affiliations:** ICMR–National JALMA Institute for Leprosy and Other Mycobacterial Diseases, Agra, India

**Keywords:** Mycobacterium leprae, leprosy, mycobacterial diseases, bacteria, Burkina Faso

**To the Editor:** Millogo et al. (*1*) documented presence of *Mycobacterium leprae* in a fecal sample from a patient in Burkina Faso, raising questions about the role of fecal excretion of *M. leprae* in the natural history and diagnosis of leprosy. They speculated that *M. leprae* were swallowed by the patient along with blood or upper respiratory secretions during leprosy rhinitis and epistaxis (*1*) but failed to address other factors that could influence fecal excretion of *M. leprae* and utility of fecal specimens in diagnosing leprosy.

Previous studies have demonstrated the presence of *M. leprae* in water and soil samples from habitations of patients with leprosy (*2*,*3*). This finding means that patients, contacts, or healthy persons can ingest *M. leprae* from environmental sources through drinking contaminated water or eating *M. leprae*–containing food and may excrete leprosy bacilli in their feces without establishing an infection. The role of environmental sources and simple pass-through phenomena in fecal excretion of *M. leprae* has not been investigated by Millogo et al. (*1*) and other studies (*4*,*5*).

Koshy et al. (*4*) reported the presence of leprosy bacilli in gastric juice of 9 of 16 patients with lepromatous leprosy; 3 were found to excrete the bacilli in their feces. Manzullo et al. (*5*) demonstrated the presence of acid-fast bacilli in biliary secretions of 7 of 20 patients with leprosy and in 2 of 7 fecal samples. These observations indicate that clinical manifestation of leprosy varies widely. The exact mechanism of fecal excretion of *M. leprae* can be more complex, as presumed in previous studies (*1*,*4*,*5*), and may be associated with high bacillary burden (as in lepromatous leprosy), gastrointestinal symptoms (abdominal pain or diarrhea), disseminated disease, environmental factors, or combinations of these aspects. Verification of transmission routes of *M. leprae* to fecal samples using genotyping techniques (i.e., whole-genome sequencing) is crucial to establish the diagnostic utility of fecal specimens in leprosy.
